# C1q/TNF-related protein 3 (CTRP3) and 9 (CTRP9) concentrations are decreased in patients with heart failure and are associated with increased morbidity and mortality

**DOI:** 10.1186/s12872-019-1117-0

**Published:** 2019-06-10

**Authors:** Chao Gao, Shasha Zhao, Kun Lian, Baibing Mi, Rui Si, Zhijun Tan, Feng Fu, Shuai Wang, Rutao Wang, Xinliang Ma, Ling Tao

**Affiliations:** 1 Department of Cardiology, Xijing Hospital, The Fourth Military Medical University, 15 Changle West Road, Xi’an, 710032 China; 2 Department of Epidemiology and Biostatistic, School of Public Health, Xi’an Jiaotong University Health Science Center, No.76, Yanta West Road, Xi’an, 710061 China; 3 Department of Statistics, The Fourth Military Medical University, 15 Changle West Road, Xi’an, 710032 China; 4 Department of Physiology and Pathophysiology, The Fourth Military Medical University, 15 Changle West Road, Xi’an, 710032 China; 5 Center for Translational Medicine, Thomas Jefferson University, Philadelphia, PA 19107 USA

**Keywords:** Biomarker, C1q/TNF-related Protein3 (CTRP3), C1q/TNF-related Protein9 (CTRP9), Heart failure with reduced ejection fraction

## Abstract

**Background:**

Biochemical marker has revolutionized the approach to the diagnosis of heart failure. However, it remains difficult to assess stability of the patient. As such, novel means of stratifying disease severity are needed. C1q/TNF-Related Protein 3 (CTRP3) and C1q/TNF-Related Protein 9 (CTRP9) are novel adipokines that contribute to energy homeostasis with additional anti-inflammatory and anti-ischemic properties. The aim of our study is to evaluate concentrations of CTRP3 and CTRP9 in patients with HFrEF (heart failure with reduced ejection fraction) and whether associated with mortality.

**Methods:**

Clinical data and plasma were obtained from 176 healthy controls and 168 patients with HFrEF. CTRP3 and CTRP9 levels were evaluated by enzyme-linked immunosorbent assay.

**Results:**

Both CTRP3 and CTRP9 concentrations were significantly decreased in the HFrEF group compared to the control group (*p* < 0.001). Moreover, patients with higher New York Heart Association class had significantly lower CTRP3 or CTRP9 concentrations. Correlation analysis revealed that CTRP3 and CTRP9 levels were positively related with LVEF% (CTRP3, *r* = 0.556, *p* < 0.001; CTRP9, *r* = 0.526, *p* < 0.001) and negatively related with NT-proBNP levels (CTRP3, *r* = − 0.454, *p* < 0.001; CTRP9, *r* = − 0.483, *p* < 0.001). After a follow up for 36 months, after adjusted for age, LVEF and NT-proBNP, we observed that CTRP3 or CTRP9 levels below the 25th percentile was a predictor of total mortality (CTRP3,HR:1.93,95%CI1.03~3.62,*P* = 0.042;CTRP9,HR:1.98,95%CI:1.02~3.85,*P* = 0.044) and hospitalizations (CTRP3,HR:2.34,95% CI:1.43~3.82,*P* = 0.001;CTRP9,HR:2.67,95%CI:1.58~4.50,*P* < 0.001).

**Conclusions:**

CTRP3 and CTRP9 are decreased in patients with HFrEF, proportionate to disease severity, and each is associated with increased morbidity and mortality.

**Trial registration:**

NCT01372800. Registered May 2011.

## Background

The discovery of novel risk markers for heart failure (HF) has contributed to improved screening, prevention, diagnosis and treatment of HF [1, 2]. Up to now, BNP and NT-proBNP are the most widely used biomarkers in clinical practice, such testing is recommended in current guidelines in determining the diagnosis and prognosis of HF. However, in certain cases, such as for extremely high BNP or NT-proBNP levels, these biomarkers cannot reflect the severity of HF or morphologic changes of the heart. Besides, increased NT-proBNP levels may reflect diminished renal function, left ventricular wall stress, or both. As such, novel means of stratifying disease severity are still needed.

Fat tissue contributes to energy homeostasis and can secrete a number of adipokines that have cardioprotective properties [3]. During heart failure, the secretion of adipokines is dysregulated [4]. However, the dysfunction of adipokines secretion in fat tissue cannot currently be easily measured. Researches have shown adiponectin level was a predictor of mortality, independent of risk markers of CHF severity. However, the levels of adiponectin in healthy people can range from 5 to 30 μg/ml [5]. It is hard to set the cutoff point between healthy and HF subjects. Interestingly, adiponectin have been recently found belonging to CTRP family [6]. The number of proteins in the CTRP family currently comprises 15 members in addition to adiponectin. Among them, in our previous in vivo studies, we found CTRP (C1q/TNF-Related Protein) 3 and CTRP9 were closely related to cardiovascular diseases [7–9].

CTRP3 is mainly expressed in mammalian subcutaneous and visceral adipocytes [10]. CTRP3 can lower blood glucose in both normal and *ob/ob* mice without affecting insulin or adiponectin levels [11]. CTRP3 is also found to be an anti-inflammatory adipokine [12] that inhibits proinflammatory pathways such as fatty acid-, LPS- and Toll-like receptor (TLR)-mediated inflammation in monocytes and adipocytes [13]. Moreover, CTRP3 promotes vascular smooth muscle cell proliferation in blood vessel wall after vascular injury [14]. CTRP9 is predominantly expressed in primary adipocytes and stromal cells [15]. CTRP9 can also reduce blood glucose and insulin levels without effecting body weight or food intake. CTRP9 is also a vasorelaxative adipocytokine that may exert vasculoprotective effects via the adiponectin receptor 1/AMPK/eNOS dependent/NO mediated signaling pathway [16]. In an in vivo MI model, we and others found CTRP3 [7] or CTRP9 [17] can improve survival rate, restored cardiac function, attenuated cardiomyocyte apoptosis, attenuated adverse remodeling, increased re-vascularization. However, to date, the levels of CTRP3 and CTRP9 have not been reported in patients with heart failure, and consequently, their possible role in relation to the severity and mortality of heart failure is unknown.

To analysis the correlation of CTRP3, CTRP9 and HFrEF, we measured levels of CTRP3 and CTRP9 in clinically controlled HFrEF patients of various degrees of severity. Further, in a 3-year follow up, we studied their association with morbidity and mortality.

## Methods

### Study population

Patients with heart failure with reduced EF that were admitted to the Department of Cardiology at Xijing Hospital, Xian, China, between May 2012 and September 2012, were consecutively recruited for this study. One hundred sixty-eight chronic heart failure patients (122 men and 46 women) were recruited. The diagnosis of HFrEF was made on the basis of clinical history, a chest roentgenogram, an electrocardiogram and an echocardiogram. The inclusion criterion was a diagnosis of heart failure (left ventricular EF < 40%). Severity of disease was assessed according to the New York Heart Association (NYHA) standards and fell within functional classes II to IV. Exclusion criteria for the study were hypotension [SBP ≤ 90 mmHg (1 mmHg = 0.133 kPa)], cardiogenic shock, severe bradycardia (resting heart rate ≤ 60 beats /min), atrioventricular block (more than II degree), ongoing acute exacerbation of heart failure, malignancy, acute systemic infections, acute or chronic liver disease or renal failure. The control group paired by age and sex consisted of 176 healthy subjects (116 men and 60 women) who underwent physical examinations, blood analysis and echocardiographic evaluations as a part of a routine health check-up in the health promotion center of Xijing Hospital between May 2012 and June 2012.

### Follow up

The study main end-points were all-cause death and the second outcomes was re-hospitalization rates. Patients were followed up by telephone once every 3 months for a minimum of 36 months. During the whole follow up period, eight patients were lost to follow up. The baseline characteristics of the patients who were lost to follow-up were not significantly different from the others, thus they were not included in the survival analyses.

### Laboratory measurements

After a minimum 8-h overnight fast, venous blood was drawn into EDTA tubes and promptly centrifuged at 4 °C, and plasma was frozen at − 70 °C for subsequent assays. Plasma glucose, total cholesterol, low-density lipoprotein and high-density lipoprotein cholesterol, triglycerides, serum creatinine, urea nitrogen, uric acid and cystatin c levels were analyzed by applying the standard protocols of the hospital biochemistry laboratory. NT-proBNP was measured by a double-antibody sandwich technique with electrochemiluminescence as signal (Elecsys 2010, Roche Diagnostics). ELISA was used for measurement of CTRP3 and CTRP9 (Shanghai LianShuo Biological Technology Co, China; The intra-assay and interassay coefficients of variation were below 5%). No significant cross-reactivity or interference between human CTRP3 and CTRP9 was observed in our pilot experiment.

### Statistical analysis

Statistical analysis was performed with the Statistical Package for Social Sciences for Windows (IBM, SPSS version 19.0) and graphs were made by Prism 6 (GraphPad software). To observe the possible difference in CTRP3/CTRP9 levels between healthy control and HFrEF patients, sample size was calculated by PASS 11 (NCSS). Sample sizes of 164 subjects in control group and 164 subjects in HFrEF group achieve 90% power to detect a difference of − 30.0 between the null hypothesis that both group means are 0.0 and the alternative hypothesis that the mean of HFrEF group is 30.0 with estimated group standard deviations of − 70.0 and 70.0 and with a significance level (alpha) of 0.01 using a two-sided two-sample t-test. All variables were tested for normality and log transformed was performed for the variables which were not non-normality.

Continuous variables are expressed as mean ± standard deviation (mean ± SD) unless otherwise stated. Categorical variables were analyzed as value and percentage. Continuous data with a normal distribution were compared with the Student t or ANOVA tests. Chi-square test was used to test for differences in the distribution of categorical variables. Correlations between CTRP3 or CTRP9 levels and other variables were evaluated by Pearson correlation coefficient analysis. Event rates were compared by Kaplan-Meier curves calculated for CTRP3 or CTPR9 above or below the 25th percentile. The independent predictive power of CTRP3 or CTRP9 and other clinical and demographic variables for death and hospitalizations for HFrEF was tested by stepwise Cox proportional hazards regression analyses. The analyses are presented as HR with a 95% confidence interval (CI). *P* values < 0.05 were considered significant.

## Results

### Baseline characteristics

The clinical and biochemical characteristics of the study subjects are presented in Table 1. At total of 168 HFrEF patients were recruited. 65% of the patients were treated with an ACE inhibitor, 36% were treated with an angiotensin II antagonist, 69% received a β-blocker, 80% were treated with digoxin and 80% were using a diuretic. The HFrEF group showed significantly increased BMI, LDL-C, triglycerides, serum creatinine and cystatin c and NT-proBNP compared to the control group. LVEF%, HDL-C and total cholesterol levels in the HFrEF group were significantly lower than in the control group. Meanwhile, age, sex, diastolic and systolic blood pressure in these two groups showed no significant differences.

**Table 1:** Clinical and biochemical characteristics of control and HFrEF groups

Variables	Control (*n* = 176)	HFrEF (*n* = 168)	*P*
Age (years)	55.11 ± 13.44	57.25 ± 14.92	0.418
Sex, male (%)	116 (65.9%)	122(72.6%)	0.199
BMI (Kg/m^2^)	21.72 ± 1.78	23.45 ± 3.80	< 0.001
Diabetes mellitus (%)	0(0%)^a^	40 (23.8%)	< 0.001
Hypertension (%)	0(0%)^a^	66 (39.3%)	< 0.001
Myocardial infarction (%)	0(0%)^a^	52 (31.0%)	< 0.001
Systolic pressure (mmHg)	108.86 ± 15.70	119.04 ± 20.71	0.071
Diastolic pressure (mmHg)	71.41 ± 10.90	75.96 ± 12.80	0.577
Fasting glucose (mmol/L)	4.72 ± 0.35	5.00 ± 1.75	0.004
HDL-cholesterol (mmol/L)	1.31 ± 0.19	0.93 ± 0.28	< 0.001
LDL-cholesterol (mmol/L)	1.67 ± 0.34	2.33 ± 0.88	< 0.001
Total Cholesterol (mmol/L)	4.16 ± 0.51	3.71 ± 0.98	0.001
Triglyceride (mmol/L)	0.95 ± 0.24	1.39 ± 0.86	< 0.001
Serum creatinine (umol/L)	91.11 ± 6.58	113.79 ± 32.89	< 0.001
Cystatin c (mg/L)	1.05 ± 0.23	1.38 ± 0.42	< 0.001
NT-proBNP (ng/L)	3.81 ± 2.55	9878.69 ± 9236.14	< 0.001
LVEF (%)	59.25 ± 2.35	28.67 ± 6.18	< 0.001
CTRP3 (ng/mL)	236.40 ± 62.86	173.30 ± 49.81	< 0.001
CTRP9 (ng/mL)	180.70 ± 51.05	124.60 ± 37.58	< 0.001

Data are expressed as mean ± SD or frequency (%)

*P-*values were calculated by an independent-samples t-test or chi-square test

*BMI* Body mass index, *HDL* High-density lipoprotein, *LDL* Low-density lipoprotein, *NT-proBNP* N-terminal pro-brain natriuretic peptide, *LVEF* Left ventricular ejection fraction, *CTRP3* C1q/TNF-related protein 3, *CTRP9* C1q/TNF-related protein 9

^a^These data were acquired by inquiry of medical history

### CTRP3 and CTRP9 levels and correlation with clinical parameters

Compared with control subjects, the concentrations of CTRP3 and CTRP9 were significantly decreased in patients with HFrEF (Table 1, *P* < 0.001, respectively). Multivariable linear regression analyses was then preformed. Results analyzed in all enrolled subjects demonstrated that Age, hypertension, NT-ProBNP, LEVF% were found to be associated with CTRP3 and CTRP9. We than set HFrEF as the outcome variable and CTRP 3/9 and other variables as predictor in a logistic model. Results were shown in Table 2. While HFrEF as the outcome variable, Age, Total Cholesterol, CTRP3 or CTRP9 exhibited statistical significance.

**Table 2:** Logistics regression analyses

	*P*	OR	95% CI. for OR			*P*	OR	95% CI. for OR	
			Lower	Upper				Lower	Upper
Age (years)	0.000	1.121	1.064	1.182	Age (years)	0.000	1.134	1.068	1.205
Sex(%)	0.246	1.965	0.628	6.146	Sex(%)	0.109	2.852	0.790	10.287
BMI (kg/m^2^)	0.509	1.061	0.890	1.265	BMI (kg/m^2^)	0.328	1.102	0.907	1.340
Serum Creatinine (μmol/L)	0.045	1.044	1.001	1.088	Serum Creatinine (μmol/L)	0.074	1.046	0.996	1.099
Cystatin C (mg/L)	0.387	0.504	0.107	2.379	Cystatin C (mg/L)	0.642	0.680	0.134	3.458
Total cholesterol (mmol/L)	0.000	0.251	0.117	0.540	Total cholesterol (mmol/L)	0.001	0.244	0.103	0.581
Triglyceride (mmol/L)	0.002	23.336	3.264	166.867	Triglyceride (mmol/L)	0.001	48.693	4.998	474.405
CTRP3 (ng/mL)	0.000	0.977	0.967	0.987	CTRP9 (ng/mL)	0.000	0.960	0.945	0.977

**Fig. 1:**
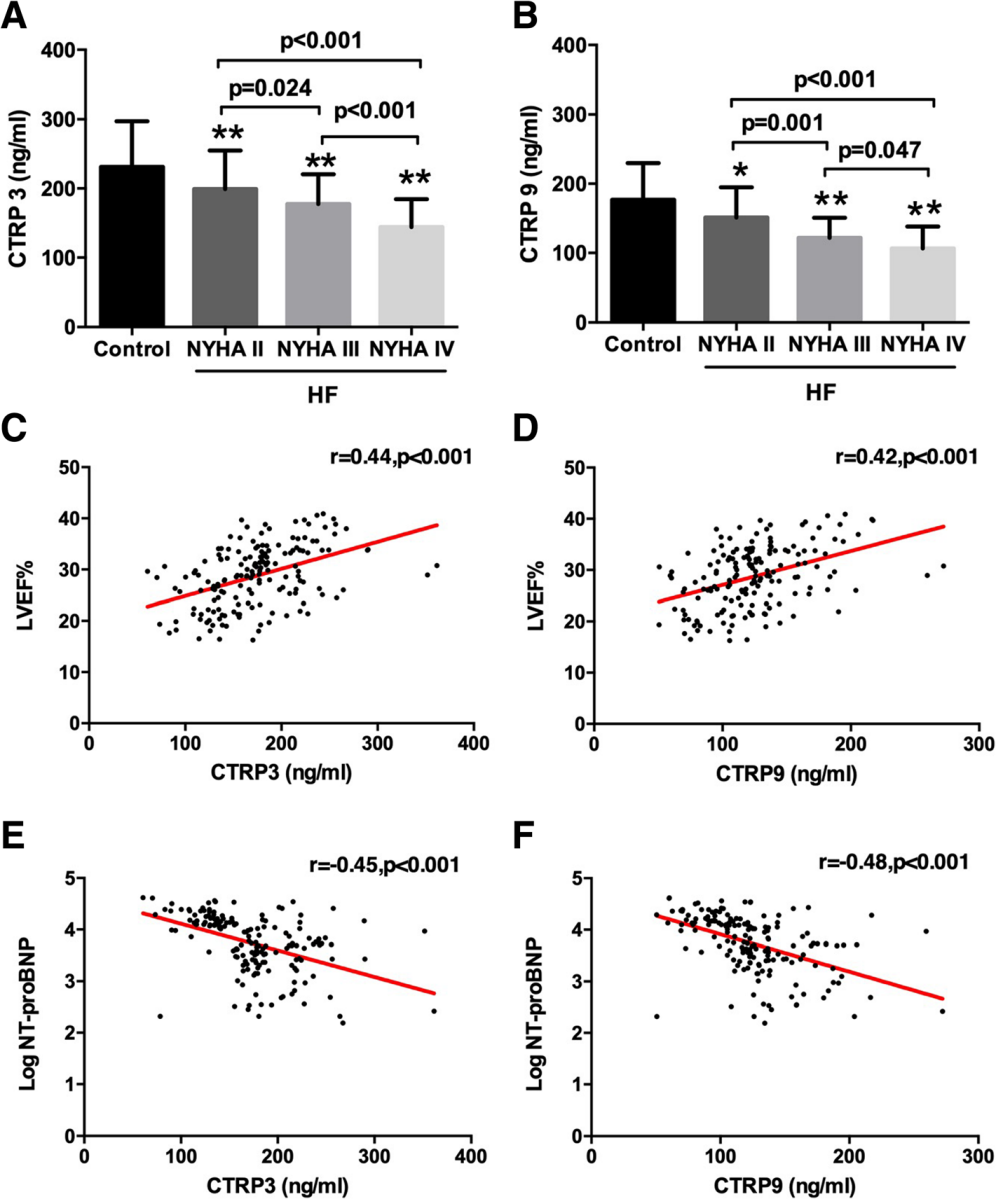
Concentration of CTRP3 (**a**) and CTRP9 (**b**) according to the severity of HFrEF stratified by NYHA functional class. Scatterplots of the association between CTRP3/CTRP9 and LVEF% (**c** and **d**) and with log transformed values of NT-proBNP (**e** and **f**) in HFrEF patients. *p < 0.05, **p < 0.01 vs. Control group

Subsequently, we analyzed relationships between CTRP3/CTRP9 and NYHA class, LVEF% and NT-proBNP in HFrEF patients. 24.4% (41 patients) of the patients with HFrEF were NYHA functional class II, 47.0% (79) were NYHA III and 28.6% (48) were NYHA IV. We found the more advanced the HFrEF symptom status according to NYHA class, the lower the CTRP3 and CTRP9 concentrations were (Fig. 1a and b). Pearson correlation analysis also revealed that in the HFrEF group, both CTRP3 and CTRP9 concentrations were positively correlated with LVEF% (CTRP3, *r* = 0.556, *P* < 0.001; CTRP9, *r* = 0.526, *P* < 0.001) and inversely associated with the log index of NT-proBNP levels (CTRP3, *r* = − 0.454, *P* < 0.001; CTRP9, *r* = − 0.483, *P* < 0.001) (Fig. 1c to f).

**Fig. 2:**
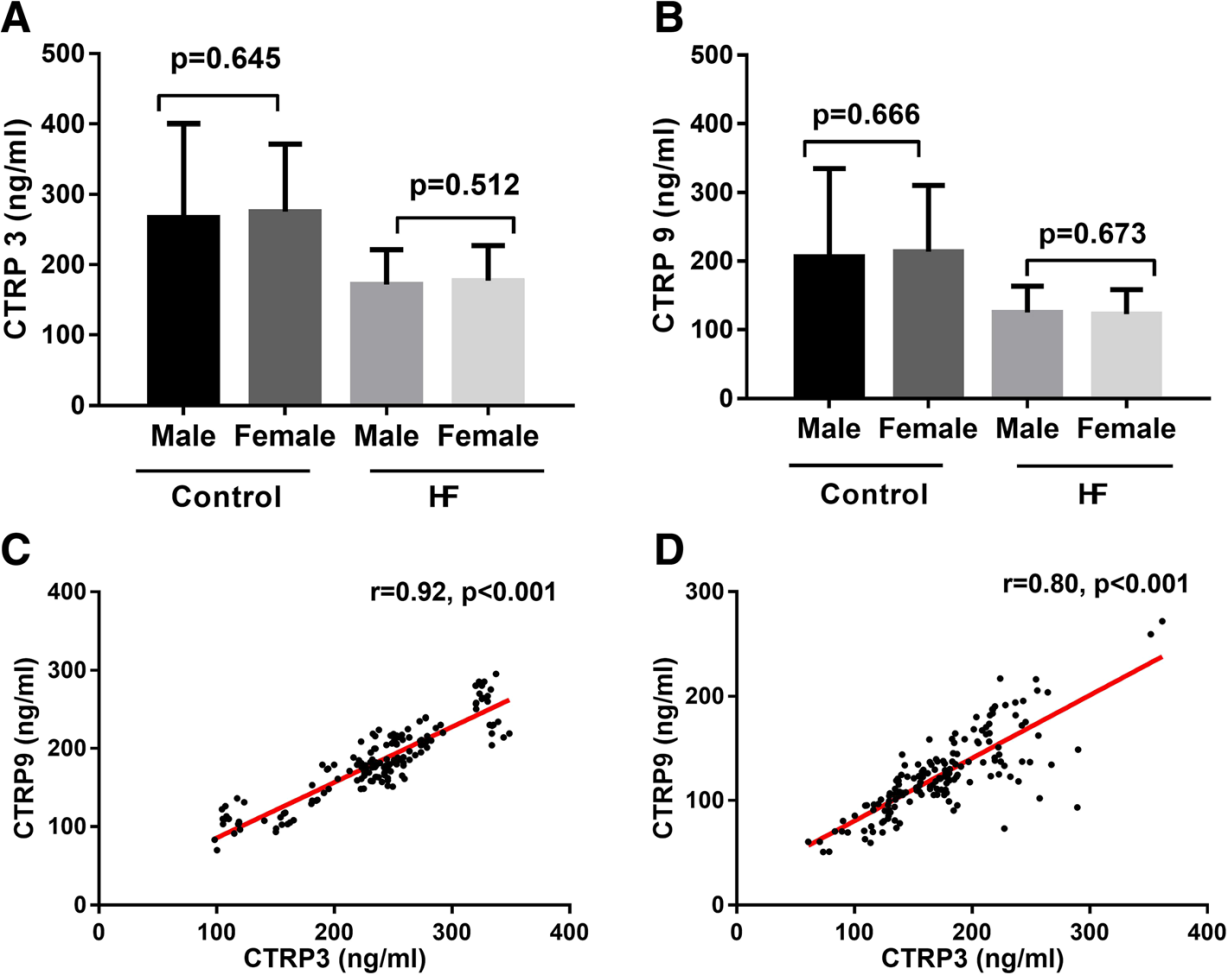
Distribution of CTRP3 and CTRP9 according to sex (**a** and **b**). Correlation between CTRP3 and CTRP9 in control group (**c**) and HFrEF group (**d**)

Most human adipose cytokines levels are affected by estrogen. To our surprise, we found that plasma CTRP3 and CTRP9 concentrations were not different according to sex in either the control group or the HFrEF group (Fig. 2a and b). To our surprise, we also observed that CTRP3 and CTRP9 concentrations manifested a significantly positive relationship in both healthy subjects and HFrEF patients (control subjects, *r* = 0.916, *P* < 0.001; HFrEF subjects, *r* = 0.800, *P* < 0.001) (Fig. 2c and d).

### Survival analyses

To investigate if lower CTRP3/CTRP9 is related with higher death rates or re-hospitalization rates in HFrEF patients, we tried to dichotomize CTRP3/CTRP9 levels for further analysis. Because CTRP3 and CTRP9 are novel adipokines and they have never been reported data showing their concentrations in heart failure patients, we dichotomize CTRP3 and CTRP9 by 25th percentile, 50th percentile and 75th percentile and tested to observe whether there are statistical significance in survive analysis. Results showed that while dichotomize CTRP3 and CTRP9 by 25th percentile, Kaplan-Meier analysis revealed differences in 3 years of follow-up in death and free-of-hospitalization above and below the first quartile of plasma CTRP3 or CTRP9. Supported by these findings, the CTRP3 and CTRP9 were dichotomize by 25th percentile for further analysis. Clinical and laboratory variables according to 25th percentile of baseline CTRP3 or CTRP9 levels in HFrEF patients are presented in Table 3. We found that patients in the lower quartile of CTRP3 levels manifested lower morbidity related to hypertension, lower rates of LVEF> 25% and lower NT-proBNP< 20,000 ng/L and surprisingly younger age. Similarly, patients in the lower CTRP9 quartile were associated with a lower morbidity of hypertension, total cholesterol and lower rate LVEF> 25% and NT-proBNP< 20,000 ng/L.

**Table 3:** Baseline characteristics according to 25 percentile of CTRP3 and CTRP9 levels

Variables	CTRP3 (ng/ml)			CTRP9 (ng/ml)		
	≤136.87 (*n* = 42)	> 136.87 (*n* = 126)	*P*	≤100.75 (*n* = 42)	> 100.75 (*n* = 126)	*P*
Age (years)	51.74 ± 19.16	59.09 ± 12.80	0.019	51.48 ± 17.58	59.17 ± 13.47	0.142
Sex,male (%)	32(76.2%)	90(71.4%)	0.549	31(73.8%)	91(72.2%)	0.842
BMI (kg/m^2^)	22.80 ± 3.86	23.66 ± 3.77	0.933	22.30 ± 3.59	23.83 ± 3.80	0.462
Diabetes mellitus (%)	7(16.7%)	33(26.2%)	0.209	6(14.3%)	34(27.0%)	0.094
Hypertension (%)	10(23.8%)	56(44.4%)	0.018	7(16.7%)	59(46.8%)	0.001
Myocardial infarction (%)	12(28.6%)	40(31.7)	0.700	9(21.4%)	43(34.1%)	0.123
Disease duration (months)	44.40 ± 56.92	33.21 ± 36.44	0.237	38.81 ± 42.59	35.07 ± 42.70	0.574
Systolic pressure (mmHg)	113.05 ± 22.334	121.03 ± 19.83	0.287	111.71 ± 20.92	121.48 ± 20.14	0.822
Diastolic pressure (mmHg)	73.45 ± 14.61	76.80 ± 12.09	0.443	71.43 ± 9.98	77.48 ± 13.30	0.021
Fasting glucose (mmol/L)	4.75 ± 1.43	5.08 ± 1.83	0.275	4.45 ± 1.10	5.18 ± 1.88	0.059
HDL-cholesterol (mmol/L)	0.86 ± 0.26	0.96 ± 0.29	0.865	0.87 ± 0.30	0.95 ± 0.28	0.486
LDL-cholesterol (mmol/L)	2.11 ± 0.84	2.40 ± 0.88	0.839	2.12 ± 0.83	2.40 ± 0.89	0.731
Total Cholesterol (mmol/L)	3.45 ± 1.00	3.80 ± 0.96	0.842	3.45 ± 0.98	3.80 ± 0.97	0.015
Triglyceride (mmol/L)	1.27 ± 0.71	1.42 ± 0.90	0.332	1.15 ± 0.56	1.47 ± 0.92	0.873
Serum creatinine (umol/L)	123.69 ± 36.954	110.49 ± 30.89	0.214	119.33 ± 34.45	111.94 ± 32.30	0.547
Cystatin c (mg/L)	1.48 ± 0.43	1.35 ± 0.42	0.428	1.40 ± 0.39	1.37 ± 0.44	0.828
NT-proBNP<20,000 (ng/L)	25 (59.5%)	121(96%)	< 0.001	27(64.3%)	119(94.4%)	< 0.001
LVEF> 25%	19 (45.2%)	103(81.7%)	< 0.001	21 (50%)	101 (80.2%)	< 0.001

**Fig. 3:**
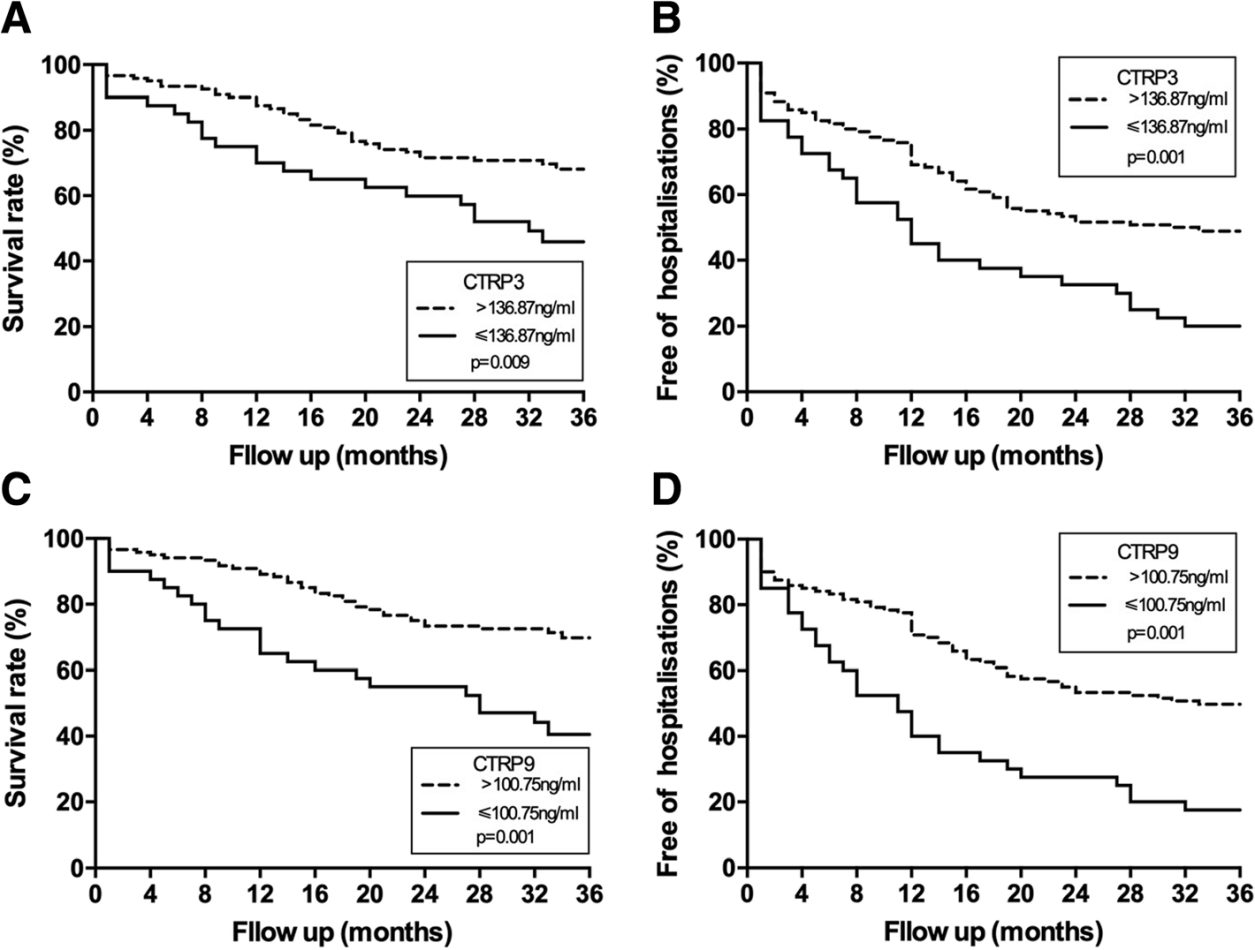
Kaplan-Meier survival curves according to the above or below 25th centile of CTRP3 or CTRP9 concentrations. **a** Event free survival rate of CTRP3. **b** Free of HFrEF hospitalization rates of CTRP3. **c** Event free survival rate of CTRP9. **d** Free of HFrEF hospitalization rates of CTRP9

During the 3 years of follow-up, 58 (36.3%) of the patients died in the HFrEF group, whereas 93 (58.1%) were hospitalized for HFrEF deterioration. Kaplan-Meier analysis revealed differences in event-free events for death and free-of-hospitalization when the group was divided according to concentrations above and below the first quartile of plasma CTRP3 (136.87 ng/ml) or CTRP9 (100.75 ng/ml) (Fig. 3). The unadjusted overall mortality risk was markedly elevated in the patients in the first quartile of CTRP3 (*P* = 0.009). This observation was in accordance with the markedly increased HFrEF hospitalization rates in these subjects (*P* = 0.001). Similarly, the unadjusted overall mortality risk and HFrEF hospitalization free rates were also markedly elevated in the subjects in the first quartile of CTRP9 (*P* < 0.001, respectively).

Multivariate analysis by stepwise Cox proportional hazards regression analysis confirmed that plasma CTRP3 concentrations (determined dichotomously, adjusted for age, hypertension, LVEF> 25%, NT-proBNP< 20,000 ng/L) remained independently predictive of death with a hazard risk ratio of 1.93 (95% CI 1.03 to 3.62, *P* = 0.042). Moreover, CTPR3 concentration was also an independent predictor of HFrEF hospitalization free rates during the follow up period with a hazard risk ratio of 2.34 (95% CI 1.43 to 3.82 *P* = 0.001). Similarly, CTRP9 (determined dichotomously, adjusted for hypertension, diabetes, LVEF> 25%, NT-proBNP< 20,000 ng/L, diastolic pressure and total cholesterol) was also found to be independently predictive of death with a hazard risk ratio of 1.98 (95% CI 1.02 to 3.85, *P* = 0.044) and a predictor of free of HFrEF hospitalization with a hazard risk ratio of 2.67 (95% CI 1.58 to 4.50, *P* < 0.001).

## Discussion

Heart failure is emerging rapidly as a major cause of morbidity and mortality around the world. As such, novel means of stratifying disease severity are needed. Here, in a prospective cohort of patients with HFrEF, we report that CTRP3 and CTRP9 levels are decreased in patients with HFrEF in a manner proportionate to symptom severity (NYHA class). Consistent with that, levels of these 2 adipokines were inversely associated with markers of HFrEF severity, such as LVEF and NT-proBNP. We also observed a significant relationship between CTRP3 and CTRP9 levels. Finally, decreased levels of CTRP3 and CTRP9 were each associated with increased mortality and hospitalization.

CTRP is a widely expressed and highly conserved adipokine family of adiponectin paralogs [18]. The number of proteins in the CTRP family is rapidly growing and currently comprises 15 members in addition to adiponectin [6]. CTRPs principally form highly stable, biologically active homotrimers and control adipocyte physiology as well as energy homeostasis [18]. In previous preclinical research, using an in vivo mouse myocardial ischemia model, we found that supplementing CTRP3 [7] or CTRP9 [9] can attenuate post-MI remodeling and augment post-MI contractile function through their anti-apoptotic and pro-angiogenic properties. However, little is known about the levels of CTRP3 and CTRP9 in patients with disease. In previous studies, data suggested that patients with acute coronary syndrome or stable angina pectoris had significantly lower plasma CTRP3 concentrations compared with control subjects [8]. In addition, previous data also showed decreased CTRP3 in obese populations [19] . No data have existed regarding CTRP9 in patients with cardiovascular disorders, but there are statistics showing that CTRP9 concentrations are decreased in patients with metabolic syndrome [20], but surprisingly elevated in obesity [21]. Although still under debate, some researches indicated that CTRP3 also share a similar pattern. CTRP3 might increase in obese subjects but decrease in T2DM patients [22–24]. In the present study, we observed that low CTRP3 or CTRP9 levels are associated with a higher likelihood of death and morbidity. Taken together with our data in previous basic research, CTRP3/CTRP9 may be a potential therapeutic and diagnostic target.

CTRP3 and CTRP9 share a similar modular organization with adiponectin [18], which is an insulin-sensitizing adipokine with anti-inflammatory and anti-atherogenic properties [25]. In the present study, we found CTRP3 and CTRP9 concentrations to be decreased in HFrEF patients, with the concentration negatively related to NYHA class. However, evidence exists that APN levels are increased in HFrEF patients [4]. In two previous cross-sectional studies, researchers found in healthy subjects, CTRP3 (*r* = 0.194, *p* < 0.001) and CTRP9 (*r* = 0.15, *p* < 0.03) are positively correlated with adiponectin, respectively. Therefore, we presume that although CTRP3, CTRP9 and APN have similar biological activities and CTRP3/CTRP9 concentration are positively related to the APN in healthy subjects, their relationship is not a causal relationship and the signaling pathways regulating the level of APN and CTRP3 and CTPR9 may be different.

Some research reported that the mRNA of CTRP3 in fat tissue, and its serum levels are similar in both sex in mice [26]. However, clinical trials showed that CTRP3 concentration was significantly higher in women than in men [27]. Similarly, an animal study examining the level of CTRP9 showed that female mice had higher levels than male mice [15]. Interestingly, contrary to the results of the animal study, in a human study, CTRP9 concentration was not different according to sex [20]. We found that in both healthy people or in patients with HFrEF, the concentration of CTRP3 and CTRP9 were not different in male and female. Our data support that CTRP3 and CTRP9 levels are similar in both sexes.

Each member in the CTRP family has its own unique biological activity [28]. However, to our knowledge, the relationships among CTPRs in the CTRP family have never been reported. The present study is the first to demonstrate that CTRP3 and CTRP9 levels have a significantly positive relationship in both healthy subjects and HFrEF patients. Previous data suggest that CTRP3 reduces tumor necrosis factor-α (TNF-α) and interleukin-6 (IL-6) secretion through suppression of nuclear factor κB signaling pathway [29]. Moreover, TNFα can inhibit CTRP9 expression via oxidative stress-mediated inhibition of transcription factor PPARγ [30]. Thus, TNF-α might be the link in regulating their levels in adipose tissue. In addition, we have reported that supplementing either CTRP3 or CTRP9 can attenuate post-MI remodeling and augment post-MI contractile function. In these experiments, whether replenishing one has an impact on the other during the MI process to exert an anti-apoptotic or pro-angiogenic effect remains unknown.

Several limitations exist in this study. First, this study enrolled a relatively small cohort of subjects from a single center. All were of Chinese ancestry. As such, generalizability to other groups of patients must be evaluated going forward. That said, our prospective study design in which we enrolled consecutive patients mitigates some of these limitations. Also, we did not measure changes in CTRP3/CTRP9 nor clinical and biochemical characteristics during follow-up, and hence, no causality of the interrelationship between these parameters can be determined. Thirdly, because this is the first study to examine CTRP3 and CTRP9 in relation to prognosis in HFrEF, the present findings should be confirmed in other studies.

## Conclusion

We demonstrate that CTRP3 and CTRP9 are each decreased in patients with HFrEF, proportionate to disease severity, and each is associated with increased morbidity and mortality.

## Data Availability

The datasets used and/or analyzed during the current study available from the corresponding author on reasonable request.

## Abbreviations

BNP: Brain natriuretic peptide
CHF: Chronic heart failure
CTRP3: C1q/TNF-related protein 3
CTRP9: C1q/TNF-related protein 9
HFrEF: Heart failure with reduced ejection fraction
NT-proBNP: N-terminal pronatriuretic peptide
